# Robust Profiling of Cytochrome P450s (P450ome) in Notable *Aspergillus* spp.

**DOI:** 10.3390/life12030451

**Published:** 2022-03-18

**Authors:** Wadzani Palnam Dauda, Peter Abraham, Elkanah Glen, Charles Oluwaseun Adetunji, Shakira Ghazanfar, Shafaqat Ali, Majid Al-Zahrani, Mawuli Kwamla Azameti, Sheik Emmanuel Laykay Alao, Afiniki Bawa Zarafi, Maryam Peter Abraham, Hannatu Musa

**Affiliations:** 1 Crop Science Unit, Department of Agronomy, Federal University Gashua, Gashua P.M.B 1005, Yobe State, Nigeria; 2 Department of Horticulture, Federal College of Horticulture, Dadin Kowa P.M.B 108, Gombe State, Nigeria; peterabraham06@yahoo.com (P.A.); marysal0915@gmail.com (M.P.A.); 3 Department of Biochemistry, Federal University Lokoja, Lokoja P.M.B 1154, Kogi State, Nigeria; elkanahglen@gmail.com; 4 Applied Microbiology, Biotechnology and Nanotechnology Laboratory, Department of Microbiology, Edo University Iyamho, Auchi P.M.B 04, Edo State, Nigeria; adetunjicharles@gmail.com; 5 National Agricultural Research Centre, National Institute of Genomics and Agriculture Biotechnology (NIGAB), Park Road, Islamabad 45500, Pakistan; shakira_akmal@yahoo.com; 6 Department of Environmental Sciences and Engineering, Government College University Faisalabad, Faisalabad 38000, Pakistan; shafaqataligill@yahoo.com; 7 Department of Biological Sciences and Technology, China Medical University, Taichung 40402, Taiwan; 8 Biological Science Department, College of Sciences and Art, King Abdulaziz University, Rabigh 80200, Saudi Arabia; maalzahrani4@kau.edu.sa; 9 Division of Molecular Biology and Biotechnology, Indian Agricultural Research Institute, New Delhi 110012, India; mawuli21@gmail.com; 10 Department of Crop Protection, Faculty of Agriculture, Ahmadu Bello University, Zaria 810107, Kaduna State, Nigeria; alaoemma@gmail.com (S.E.L.A.); azarbaw@gmail.com (A.B.Z.); 11 Department of Botany, Ahmadu Bello University, Zaria 810107, Kaduna State, Nigeria; hannatumusa23@gmail.com

**Keywords:** *Aspergillus* spp., fungi, P450s, phylogeny, secondary metabolite

## Abstract

Cytochrome P450s (P450ome) constitute an extended superfamily group of heme-thiolate enzymes identified in all biological domains. P450omes play a critical role in the oxidation of steroids and fatty acids, xenobiotic degradation of hydrophobic compounds, biosynthesis of hormones, and primary and secondary metabolism in organisms. *Aspergillus* species are among the most economically important fungal organisms in human medicine, industry, and agriculture worldwide. Exploring insight on the genome-wide annotations of cytochrome P450s in *Aspergillus* species is necessary for their biosynthetic applications. In this present study, we report the identification of 306 cytochrome P450s and their robust profiling in eight notable *Aspergillus* species (*A. carbonarius*, *A. clavatus*, *A. flavus*, *A. fumigatus*, *A. nidulans*, *A. niger*, *A. oryzae,* and *A. terreus*). Based on the evolutionary relationship, the *Aspergillus* P450s families clustered into 15 clades, with clades V, I, and XIII recording higher percentages (17.3%, 15.00%, and 14.71%, respectively) of Cyp families. Cyps were classified into 120 families 64 clans, and their putative functions were also elucidated. P450s were predicted to be located in 13 subcellular components, but the endoplasm reticulum was the dominant location across the eight *Aspergillus* species. Cyps genes of *Aspergillus* species were associated with seven secondary metabolism-related gene clusters. Elucidating the genome-wide annotations of P450s enzymes in *Aspergillus* species will form vital potential biotechnological tools that could be harnessed for industrial, pharmaceutical, and agricultural use.

## 1. Introduction

*Aspergillus* is the most extensively studied genus in the fungal kingdom, with 350 identified species so far [1,2]. This holds because of the ubiquitous nature, wide host range, and principally, the economically important roles of *Aspergillus* species in the medical, industrial, and agricultural sectors globally [3,4]. In medicine, for example, invasive Aspergillosis induced by *Aspergillus* spp., especially *A. fumigatus* and *A. terreus,* are the major causes of death in patients with compromised immunity [5], where rates of mortality in patients with acute leukemia are as high as 50%, thereby making the disease to be considered as the most important human fungal infection [6]. In agriculture, *Aspergillus* spp. (*A. cabonarius*, *A. flavus*, *A. niger*, *A. ochraceus,* and *A. parasiticus*,) infect and produce mycotoxins, such as aflatoxins (aflatoxin B1, B2, G1, and G2), Sterigmatocystin, and ochratoxins, which contaminate wide varieties of agricultural produce/products, such as nuts (walnut, almond, brazil nut, pistachios, groundnuts) dried fruits, cereal/pulse grains (wheat, maize, millet, rice cocoa, and coffee beans), and grapes during pre-harvest or post-harvest periods [7]. Direct consumption of mycotoxin contaminated food produce (primary mycotoxicosis) by humans and animals or feeding on contaminated animal products (secondary mycotoxicosis) by humans have been implicated in posing serious carcinogenic, toxic, or lethal effects worldwide [8,9]. The global industrial application of some *Aspergillus* spp. has been noteworthy. *Aspergillus oryzae,* for instance, has been notably certified as a safe fungal species in the synthesis of various industrial enzymes for use in the food-processing industries [10,11]. Several species of *Aspergillus* have been found to possess the Alfa-glucuronidase enzyme, which is an essential biocatalyst in the biotransformation of lignocellulosic agro-industrial wastes into biofuels [12]. Filamentous fungi species, especially *A. niger*, among other microorganisms, are known to synthesize phytases enzyme, a form of phosphatases that is used as additives in animal feed and for production of ethanol in food and chemical industry, while the alkaline phosphatases, an enzyme derived from *Aspergillus* species, such as *A. fumigatus,* were reported to be an indispensable biotechnological tool for probing of nonisotopic, nucleic acids dephosphorylation, enzyme-linked immunoabsorbent assays (ELISA), and labelling of proteins [13,14]. Cytochrome P450 monooxygenases are distinct multigenic enzymes well known as versatile catalysts of a wide range of biochemical reactions, such as biosynthesis of primary and secondary metabolites in all life systems [15]. The key roles played by CYP protein genes in fungal species, which majorly influence their ubiquitous environmental adaptation and global impact in various economic sectors by enzymatic catalyzing the degradative and oxidative pathways of plethora aliphatic and aromatic hydrocarbon as well as other xenobiotic compounds, have been well documented [15,16,17,18,19]. Given the varying and significant functions regulated by CYP450s in different organisms and availability of their enriched databases, the CYPome of several important fungal species, such as *Phanerochete chrysosporium* [20], *Mycosphaerella graminicola* [21], *Grosmannia clavigera* [22], *Trichoderma* spp. [23], *Cunninghamella elegans* [24], and *Fusarium* species [25], have been comprehensively studied.

However, comparative evolutionary studies among *Aspergillus* species based on their CYPome are still lacking. Therefore, this present study elucidated and compared the CYP450s evolutionary descents, possible family and clan expansion, putative functions, subcellular localization, and secondary metabolite-related gene clusters among some important *Aspergillus* species.

## 2. Materials and Methods

### 2.1. Selection of Aspergillus Species, Sequence Retrieval, and Validation

A total of eight *Aspergillus* species (*A. carbonarius* ITEM 5010 v3, *A. clavatus* NRRL 1, *A. flavus* NRRL3357 v1.0, *A. fumigatus* A1163, *A. nidulans* A1146, *A. niger* van Tieghem ATCC 13496 v1.0, *A. oryzae* RIB40, and *A. terreus* NIH 2624) were selected for the present study based on their sequences availability in the MycoCosm (http://genome.jgi-psf.org/programs/fungi/index.jsf, accessed on 18 April 2021) and presence of CYP450 conserved domain in the Fungal Cytochrome P450 database (FCPD) (http://p450.riceblast.snu.ac.kr/blast.php, accessed on 3 May 2021). The protein gene sequences (1576) of the eight *Aspergillus* spp. (Supplementary file S1) were retrieved from the Joint Genome Institute (http://genome.jgi-psf.org/programs/fungi/index.jsf, accessed on 18 April 2021), out of which a total of 306 sequences were considered valid for further analyses (Supplementary files S2 and S3) in the present study after screening them for the presence of Cyp450 conserved domain (CD) in the NCBI batch CD database with a high stringency e-value of ≥7 [25].

### 2.2. Identification of CYP450 Protein Gene Families and Clans in Eight Aspergillus Species

The CYP450 families and clans of each *Aspergillus* spp. were determined by blasting their respective cyp protein gene sequences in the FCPD (http://p450.riceblast.snu.ac.kr/blast.php, accessed on 3 May 2021) against the cyp sequences of all characterized fungal species available in the database at 1e–5e-value, and the queried sequences were assigned families of their corresponding fungal species at a homology value of ≥40% as a CYP450 family classification criterium approved by the International P450 Nomenclature Committee [23]. The cyp families of each *Aspergillus* spp. were then used to locate their respective clans in the FCPD (http://p450.riceblast.snu.ac.kr/clans.php, accessed on 3 May 2021).

### 2.3. Evolutionary Relatedness of Cyp Protein Genes Amongst Eight Aspergillus Species

This analysis was perfumed in the MEGA X software package (http://www.megasoftware.net/, accessed on 3 May 2021), in which the MUSCLE algorithm was employed for the multiple sequence alignment of 306 protein gene sequences. The sequences alignment and phylogenetic tree construction specifications followed as described by Dauda et al. [26].

### 2.4. Prediction of Subcellular Localization of Cyp Protein Genes

Two online servers (http://www.jci-bioinfo.cn/iLoc-Animal, accessed on 3 May 2021) and (http://busca.biocomp.unibo.it/, accessed on 3 May 2021) were explored and harmonized for the prediction of both single and multiple subcellular localization of cyp protein genes in the queried *Aspergillus* species.

### 2.5. Gene Clusters Associated with Secondary Metabolite Synthesis

Gene clusters responsible for the biosynthesis of secondary metabolites in the eight *Aspergillus* species were annotated and retrieved from the Joint Genome Institute (https://jgi.doe.gov/our-science/science-programs/secondary-metabolites/, accessed on 18 April 2021).

## 3. Results

### 3.1. Diverseness and Widespread of Cyp450 Families and Clans in Eighth Aspergillus Species

**Figure 1:**
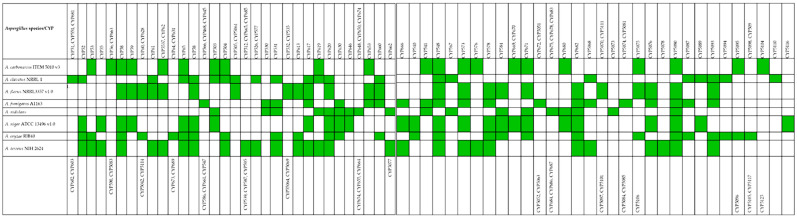
Various cytochrome P450 families predicted in eight *Aspergillus* species. NB: Green-colored boxes indicate the predicted CYP families in each *Aspergillus* species.

The results in Table 1 and Table 2 and Figure 1 present the annotations, classification, and distribution of eight *Aspergillus* species drawn from their complete Cyp proteins genes ranging from 48 to 30, with *A. oryzae* and *A. nidulans* having the highest (48) and lowest (30) number of cyp proteins, respectively. The results show that the queried *Aspergillus* species were identified in a total of 120 families and 64 clans of CYP450 (Supplementary file S1). The highest number (28) of CYP450 clans was identified in *A. flavus*, and the least (19) was found in *A. niger*. *A. oryzea* is shown to possess the highest number (37) of the family, followed by *A. flavus* (36), *A. terreus* (35), *A. fumigatus* (31), *A. cabonarius* and *A. nidulans* (29), and *A. clavatus* (28), while *A. niger* had the lowest number (26) of Cyp family. However, none of the Cyp family was identified in all the *Aspergillus* species. Five *Aspergillus* species were annotated to share nine Cyp families, namely Cyp58 (*A. cabonarius*, *A. flavus*, *A. niger*, *A. oryzea,* and *A. terreus*); Cyp56 (*A. cabonarius*, *A. clavatus*, *A. niger*, *A. oryzea,* and *A. terreus*); Cyp68 (*A. clavatus*, *A. flavus*, *A. oryzea*, *A. terreus,* and *A. niger*); Cyp531 (*A. clavatus*, *A. fumigatus*, *A. nidulans*, *A. oryzea,* and *A. terreus*); Cyp617 (*A. clavatus*, *A. flavus*, *A. fumigatus*, *A. terreus,* and *A. niger*); Cyp673 (*A. cabonarius*, *A. flavus*, *A. fumigatus*, *A. niger,* and *A. terreus*); Cyp578 (*A. flavus*, *fumigatus*, *A. niger*, *A. oryzea,* and *A. terreus*); Cyp671 (*A. cabonarius*, *A. flavus*, *A. nidulans*, *A. niger,* and *A. oryzea*);, and Cyp5093 (*A. clavatus*, *A. flavus*, *A. fumigatus*, *A. terreus* and *A. oryzea*). Each of the queried species was elucidated to have more than one Cyp family unique to them except for *A. niger* (having just one unique family Cyp5116). A total of 14 families (Cyp566, Cyp468, Cyp645, Cyp586, Cyp661, Cyp547, Cyp672, Cyp5051, Cyp5052, Cyp5063, Cyp5074, Cyp5081, Cyp5084, and Cyp5085) were detected to be exclusively found in *A. fumigatus*. This is followed by *A. nidulans,* with 12 unique families (Cyp648, Cyp650, Cyp674, Cyp654, Cyp655, Cyp664, Cyp675, Cyp678, Cyp683, Cyp684, Cyp686, and Cyp5073), while eight families each were found in *A. flavus* (Cyp532, Cyp535, Cyp55064, Cyp5069, Cyp5070, Cyp5111, Cyp5097, and Cyp5101) and *A. oryzea* (Cyp64, Cyp631, Cyp673, Cyp689, Cyp5098, Cyp509, Cyp5105, and Cyp5117); *A. terreus* has seven unique families (Cyp512, Cyp665, Cyp685, Cyp539, Cyp587, Cyp595, and Cyp5078), while six unique families were predicted in *A. clavatus* (Cyp51, Cyp551, Cyp681, Cyp602, Cyp603, and Cyp5110) and *A. cabonarius* (Cyp56, Cyp663, Cyp590, Cyp5083, Cyp5104, and Cyp5125) each (Table 2).

### 3.2. Phylogenetic Distribution of Eight Aspergillus spp. in Cytochrome P450 Families and Clans

**Figure 2:**
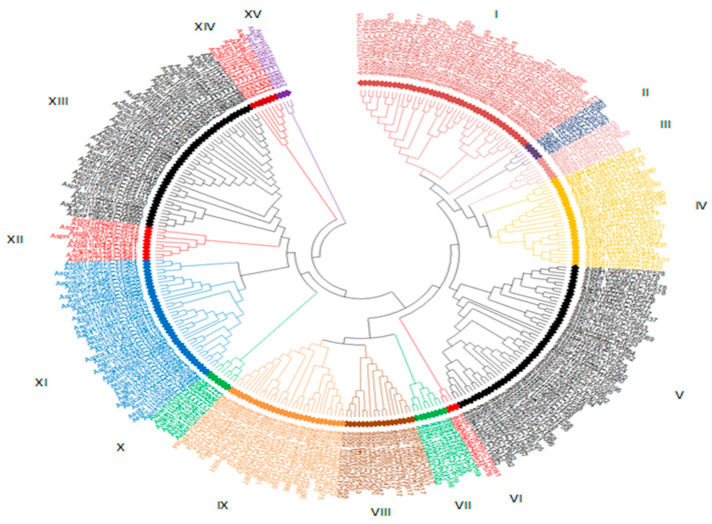
Evolutionary relationships of cytochrome P450 proteins among eight *Aspergillus* species (*A. carbonarius*, *A. clavatus*, *A. flavus*, *A. fumigatus*, *A. nidulans*, *A. niger*, *A. oryzae*, and *A. terreus*). Phylogenetic tree was inferred using the minimum evolution method with MEGAX software. Each phylogenetic group (I–XV) is indicated by a specific color.

Figure 2 showcase the evolutionary relatedness of Cyp proteins amongst eight *Aspergillus* species where their annotated families were distributed across 15 phyletic clades. 

The phyletic distribution of the Cyp proteins amongst eight *Aspergillus* species with their annotated families and clans, specific functions and respective number of protein sequences in each clade are presented in Table 3.

Results from Figure 2 and Table 3 have shown that; phyletic group 1 has the highest number of cyp protein entries (46), with all the eight *Aspergillus* species present and distributed in 15 families and seven clans performing both primary and secondary metabolism functions. Group 1 is observed to be further subdivided into three sub-phyletic clades. A total of 29 cyp protein genes are closely clustered to form subclade 1 with 9 and 5 (CYP65, CYP548, CYP672, CYP566, and CYP5083) cyp families and clans, respectively. In FCPD, 4 out of the 11 Cyp families of Clan CYP65 were in *Aspergillus* spp. These families include Cyp60, having two cyp genes, one each identified in *A. flavus* and *A. oryzae*; Cyp65 family, which contains the highest number (12) of genes in this subclade with four cyp genes identified in *A. carbonarius* and two genes each in *A. clavatus*, *A. niger*, *A. oryzae,* and *A. terreus*; Cyp567 has two cyp genes one each identified in *A. clavatus* and *A. nidulans*; and Cyp5117, which possesses a cyp gene in *A. oryzae*. A total of 15 cyp genes from all the eight *Aspergillus* spp. clustered within a single clan CYP574 to constitute subclade 2. The cyp genes were distributed in five (Cyp671, Cyp628, Cyp670, Cyp669, and Cyp5076) out of seven families of Clan CYP547. Cyp671 contains five cyps (each identified in *A. flavus*, *A. carbonarius*, *A. nidulans*, *A. niger*, and *A. oryzae*); four cyp genes in Cyp5076 family were identified each in *A. niger*, *A. flavus*, *A. fumigatus,* and *A. terreus*. Cyp670 and Cyp669 contain two genes each found in A. *niger* and A. *carbonarius*, while Cyp628 family has two Cyps, each located in *A. flavus* and *A. oryzae*. Subclade III of group I consists of only two Cyp protein clusters from Cyp578 and Cyp671 identified in *A. oryzae* and *A. niger*, respectively, and two clans (CYP578 and CYP574).

Total of five cyp protein genes from four *Aspergillus* species forms the phyletic group II, which includes two cyp protein genes from *A. flavus* and one each from *A. oryzea*, *A. niger,* and *A. fumigatus,* clustered to form phyletic group II

A total of five cyp protein genes from five clans (CYP5084, CYP58, CYP65, CYP673, and CYP507) clustered to form phyletic group II. CYP55084 clan has two families (Cyp5121 and Cyp5084), but only one cyp gene from Cyp5084 family was identified in *A. fumigatus,* while a Cyp protein from the only family in CYP673 clan was found in *A. oryzea*. Of the 14 families in CYP58 clan, only a protein gene from Cyp680 family was located: *A. niger*. Two cyps were identified in *A. flavus* from Cyp535 and Cyp567 families in CYP507 and CYP65 clans, respectively.

Clade III constitutes the clustering of seven cyp genes proteins from two families, (Cyp 630 and Cyp53) with four and three cyps genes positioned in Cyp630 (*A. oryzea*, *A. niger*, *A. nidulans,* and *A. fumigatus*) and Cyp53 (*A. oryzae* (1) and *A. terreus* (2)), respectively.

Clade IV is made up of a 27-cyp-protein gene cluster from 10 Cyp families (Cyp573, Cyp5080, Cyp5104, Cyp675, Cyp674, Cyp531, Cyp5077, Cyp631, Cyp5078, and Cyp532). The clade was subdivided distinctively in to three subgroups: in subgroup 1, five cyp genes are each located from *A. fumigatus*, *A. flavus*, *A. niger*, *A. terreus,* and *A. cabonarius,* all located within Cyp537 family. Ten cyp genes from four Cyp families (Cyp5080, Cyp5104, Cyp675, and Cyp674) formed subgroup 2, where Cyp5080 contained seven genes (one each from *A. fumigatus*, *A. flavus*, *A. niger*, *A. terreus*, and *A. oryzae* and two from *A. cabonarius*), while Cyp5104 (*A. cabonarius*), Cyp675(*A. nidulans*), and Cyp674 (*A. nidulans*) families share one cyp gene each. A total of 12 cyp genes clustered from five families (Cyp531, Cyp532, Cyp5078, Cyp631, and Cyp5077) constituting subgroup 3, in which the Cyp531 family dominated with seven genes from five *Aspergillus* spp. (*A. clavatus* (2), *A. oryzae* (2), *A. nidulans* (1), *A. fumigatus* (1), and *A. terreus* (1)); Cyp5077 had two genes (one each in *A. nidulans* and *A. terreus*), and there is one cyp gene each from Cyp532 (*A. flavus*), Cyp5078 (*A. terreus*), and Cyp631 (*A. oryzae*).

Clade V recorded the highest number (53) of cyp protein-gene clusters, with all the seven *Aspergillus* species present and distributed in 20 Cyp families (Cyp578, Cyp577, Cyp537, Cyp683, Cyp5089, Cyp53, Cyp62, Cyp684, Cyp678, Cyp5064, Cyp680, Cyp5095, Cyp5096, Cyp682, Cyp551, Cyp5094, Cyp58, Cyp681, Cyp5105, and Cyp5097). Three subclades’ clusters were also identified in Clade V. The first subclade is a cluster of 21 Cyp gene proteins in eight families, where Cyp578 dominates with seven cyps from four *Aspergillus* spp., (*A. fumigatus* (1), *A. flavus* (2), *A. terreus* (2), and *A. niger* (2)), followed by Cyp537 with four cyps (*A. cabonarius* (2), *A. terreus* (1), and *A. flavus* (1)), Cyp62 with three cyps (*A. flavus*, *A. cabonarius*, and *A. terreus*), and Cyp5089 (*A. clavatus* and *A. niger*) and Cyp577 (*A. terreus* and *A. clavatus*) with two cyps each, while Cyp684 (*A. nidulans*), Cyp53 (*A. cabonarius*), and Cyp683 (*A. niger*) had one cyp gene each.

A single cyp protein gene of *A. nidulans* from Cyp678 family constitutes subclade 2 in Clade V. However, eleven families with a total of 31 cyp genes spread across seven *Aspergillus* species and clustered to form the third subclade in Clade V. Cyp58 family contributed the highest number (10) of cyps from five *Aspergillus* spp., (*A. oryzae* (3), *A. terreus* (3), *A. niger* (2), *A. flavus* (1), and *A. cabonarius* (1)), which is followed by Cyp682 with seven cyp genes from six *Aspergillus* spp., (two cyps in *A. nidulans* and one cyp each in *A. niger*, *A. oryzae*, *A. fumigatus*, *A. terreus*, and *A. flavus*), while five families, namely Cyp5064 (*A. flavus*), Cyp681 (*A. clavatus*), Cyp5097 (*A. flavus*), Cyp551 (*A. clavatus*), and Cyp5105 (*A. oryzae*), had one cyp gene each.

Three cyp genes clustered closely from Cyp5081 (*A. fumigatus*) and Cyp576 (*A. cabonarius* and *A. terreus*) families to form Clade VI. 

Eight cyp genes from six *Aspergillus* spp. and distributed in four families (Cyp59, Cyp587, Cyp586, and Cyp662) clustered as Clade VII. Four and one cyp genes from Cyp59 (*A. cabonarius* (1), *A. flavus* (2), and *A. niger* (1)) and Cyp587 (*A. terreus*) families form subgroup 1, while subgroup 2 constitutes two cyp genes from Cyp662 (*A. terreus* and *A. nidulans*) and a gene from Cyp586 (*A. fumigatus*) families.

Clade VIII is a cluster of 17 cyp protein genes from six *Aspergillus* species and families (Cyp617, Cyp547, Cyp5070, Cyp526, Cyp540, and Cyp468) with two identified subdivisions and each sharing three families. Eight of eleven cyp genes in subgroup 1 are domiciled in Cyp617 family from five *Aspergillus* spp. (*A. niger* (2), *A. clavatus* (2), *A. fumigatus* (2), *A. terreus* (1), and *A. flavus* (1)), while Cyp547 (*A. fumigatus* and *A. terreus*) and Cyp5070 (*A. flavus*) families had two and one cyps, respectively. Cyp540 (*A. niger*, *A. oryzae,* and *A. terreus*), Cyp526 (*A. terreus* and *A. clavatus*), and Cyp468 (*A. fumigatus*) families constitute subgroup II with three, two, and one cyps, respectively.

Clade IX showed a close association of 30 cyp entries from eight *Aspergillus* spp. across 14 Cyp families (Cyp5075, Cyp5106, Cyp590, Cyp548, Cyp666, Cyp56, Cyp5099, Cyp661, Cyp655, Cyp5087, Cyp52, Cyp5051, Cyp539, and sCyp584). The clade further differentiated into three separate subclades. Subclade 1 involve fourteen members belonging to Cyp5075 (*A. flavus* (2), *A. oryzae* (2), and *A. cabonarius* (1)), Cyp666 (*A. terreus*, *A. niger*, *A. oryzae,* and *A. fumigatus*), Cyp5106 (*A. cabonarius*), Cyp56 (*A. cabonarius)*, Cyp5099 (*A. oryzae*), Cyp590 (*A. cabonarius)*, and Cyp661 (*A. fumigatus*) families. Only Cyp655 family constitutes subclade 2, with one cyp gene from *A. nidulans*. In subgroup 3, six families were identified with 15 entries, out of which five, four, and three were found in Cyp52 (*A. terreus* (2), *A. oryzae*, *A. clavatus,* and *A. niger*), Cyp584 (*A. flavus*, *A. nidulans*, *A. oryzae,* and *A. niger*), and Cyp5087 (*A. fumigatus*, *A. clavatus,* and *A. oryzae*) families, respectively, while Cyp5051 (*A. fumigatus*), Cyp548 (*A. terreus*), and Cyp539 (*A. terreus*) families contain one cyp each. Three families (Cyp613, Cyp686, and Cyp685) having seven cyp protein genes made up Clade X, which separated into two groups. Two cyp genes of *A. terreus* from Cyp685 family and a cyp gene of *A. nidulans* from Cyp686 differentiated as subgroup 1, while subgroup 2 is a cluster of four Cyps from Cyp613 (*A. oryzae* (2), *A. flavus,* and *A. terreus*) family. Xenobiotic metabolism was predicted as the major activity of the cyp members in Clade X.

A total of 38 proteins genes present across all the eight *Aspergillus* spp. from 16 families are members of clade XI. There exist three subgroups. Five protein entries, all in Cyp504 (*A. cabonarius* (2), *A. clavatus*, *A. terreus,* and *A. oryzae*), cluster as subgroup 1. Subgroup 2 consists of eight proteins from Cyp620 (*A. oryzae* (2), *A. niger* (2), *A. flavus* (2), *A. terreus,* and *A. nidulans*) and a protein gene from Cyp64 (*A. oryzae*).

Clade XII is a cluster of ten cyp protein genes from four *Aspergillus* spp. and four families, which are further divided into two subphyletic groups. Cyp51, having two cyps from *A. clavatus,* formed subgroup 1, while five, two, and one Cyps from Cyp61 (*A. terreus* (3), *A. clavatus,* and *A. flavus*), Cyp55 (*A. niger* and *A. terreus*), and Cyp5116 (*A. niger*) families, respectively, constituted subgroup II. 

Clade XIII shows the close relatedness of 45 cyp genes across seven *Aspergillus* species, having the highest number (26) of P450 families (Cyp660, Cyp5090, Cyp5101, Cyp5111, Cyp5100, Cyp5106, Cyp646, Cyp5110, Cyp653, Cyp503, Cyp698, Cyp602, Cyp5093, Cyp654, Cyp5125, Cyp503, Cyp512, Cyp5085, Cyp648, Cyp603, Cyp595, Cyp68, Cyp5061, Cyp5074, Cyp5073, and Cyp650) with two distinct subdivisions. A total of 21 cyp families are contained in subgroup I, with Cyp68 family contributing the highest number (seven) of cyp genes from *A. niger* (2), *A. oryzae* (2), *A. clavatus,* and *A. flavus*. Cyp653 family contained three cyp genes from *A. cabonarius*, *A. flavus,* and *A. fumigatus*. Cyp5061 (*A. clavatus* and *A. flavus*), Cyp503 (*A. clavatus* and *A. niger*), Cyp5106 (*A. flavus* and *A. oryzae*), Cyp646 (*A. nidulans* and *A. niger*), and Cyp5093 (*A. clavatus* and *A. flavus*) families had two cyp genes each, while Cyp5074 (*A. fumigatus*), Cyp5073 (*A. nidulans*), Cyp650 (*A. nidulans*), Cyp603 (*A. clavatus*), Cyp595 (*A. terreus*), Cyp512 (*A. terreus*), Cyp5085 (*A. fumigatus*), Cyp698 (*A. oryzae*), Cyp602 (*A. clavatus*), Cyp654 (*A. nidulans*), Cyp5125 (*A. cabonarius*), and Cyp5110 (*A. clavatus*) each contributed a cyp protein gene in the subgroup. Ten cyp genes within five families made up subgroup II, where Cyp660 family dominated the subgroup, having six cyps from *A. flavus* (2), *A. oryzae* (2), *A. clavatus,* and *A. fumigatus,* while Cyp5090 (*A. fumigatus*), Cyp5101 (*A. flavus*), Cyp5111 (*A. flavus*), and Cyp5100 (*A. oryzae*) contributed one cyp gene each.

Clade XIV presents the relationship among seven cyp genes of six *Aspergillus* species from families Cyp541 (*A. fumigatus*, *A. terreus*, *A. cabonarius,* and *A. nidulans*) and Cyp505 (*A. clavatus* (2) and *A. flavus* (1)), predicted to perform primary metabolic activity.

Clade XV is the phyletic group with the least number (three) of Cyp genes within three *Aspergillus* species and from three families, namely Cyp687 (*A. nidulans*), Cyp645 (*A. fumigatus*), and Cyp682 (*A. flavus*).

### 3.3. Prediction of Subcellular Localization Analysis of CYP450 Protein Genes of Eight Aspergillus Species

**Figure 3:**
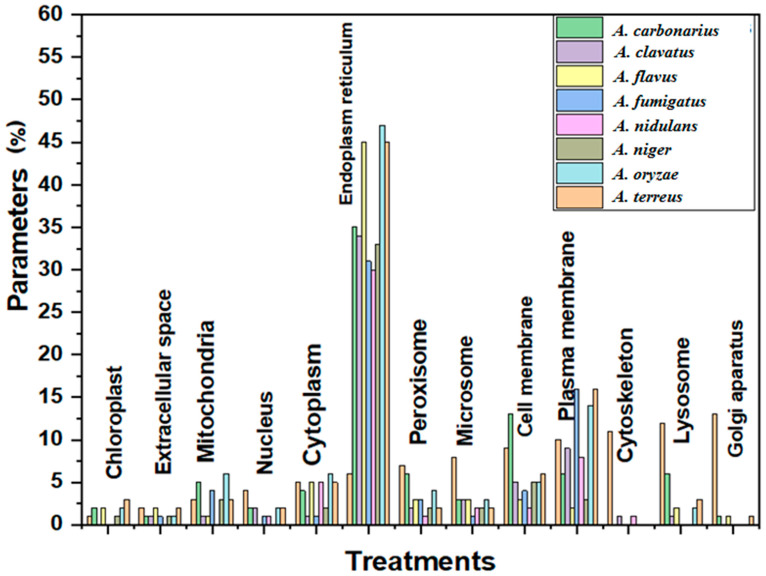
Prediction of subcellular localization analysis of protein genes of eight *Aspergillus* species.

The result of the analysis of subcellular localization of Cyp protein genes in eight *Aspergillus* spp. is presented in Figure 3. Cyp protein genes of *Aspergillus* species have been predicted to be localized in 13 subcellular organelles. However, Cyps of the queried *Aspergillus* species were not distributed across all the 13 organelles. The Cyps protein genes of *A. cabonarius* and *A. terreus* were the most widely distributed been localized across all the subcellular components except the cytoskeleton. A total of 11 organelles were predicted to contain at least one Cyp protein gene of *A. clavatus*, *A. flavus*, and *A. oryzae*. All the queried *Aspergillus* spp. were domiciled in six organelles (cytoplasm, endoplasm reticulum, peroxisome, microsome, cell membrane, and plasma membrane). Amongst the predicted organelles, the endoplasm reticulum dominates, having the highest frequency of cyp protein genes across the *Aspergillus* species. This is followed by plasma and cell membranes, while the Golgi apparatus had the least dominant subcellular structure.

### 3.4. CYP450s Implicated with Secondary Metabolism-Related Gene Clusters in Aspergillus Species

**Figure 4:**
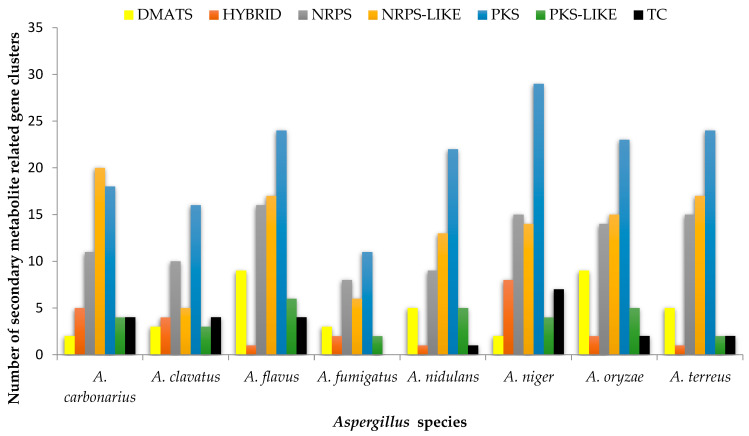
Secondary Metabolism-Related Gene Clusters for the Eight *Aspergillus* Species.

The results of the analysis in Figure 4 show the involvement of cyp protein genes of *Aspergillus* species in seven secondary metabolism-related gene clusters, namely DiMethyl Allyl Tryptophan Synthase (DMATS), Hybrid, Non-Ribosomal Peptide Synthetases (NRPS), Non-Ribosomal Peptide Synthetases-LIKE (NRPS-LIKE), PolyKetide Synthases (PKS), PolyKetide Synthases-LIKE (PKS-LIKE), and Terpene Cyclases (TC). CYP450 gene clusters for secondary metabolism were present in all our queried *Aspergillus* spp. except for TC in *A. fumigatus*. PKS was identified as the most dominant secondary metabolism-related gene cluster across the studied *Aspergillus* species, with an average of 21 cyps. NRPS-LIKE was the next-dominant secondary metabolism-related gene cluster, having an average of 13 cyps, while HYBRID and TC were the least, with three as the average number of cyp proteins. *Aspergillus niger* was predicted to have the highest number (79) of cyp genes engaged in secondary metabolic activity. This is followed by *A. flavus* (77), while *A. fumigatus* had the least (32).

## 4. Discussion

P450 genome-wide comparisons and annotations have helped us to establish better the links among Cyp families in various *Aspergillus* species. A comprehensive phylogenetic analysis was performed to illustrate the divergence of primary sequences and evolutionary connections of cytochrome P450 groups in *Aspergillus*. The phylogenetic investigations have revealed that the magnitude of the distribution of Cytochrome P450 throughout the fifteen clades differs. In evolutionary terms, the CYPome (the total number of CYPs in a particular species) is very dynamic, and the families, subfamilies, and fraction of CYP genes belonging to each of the eight *Aspergillus* species vary substantially. These distinctions are reflected in qualitative and quantitative differences in metabolite profiles and physical or developmental traits and adaption to specific ecological niches [27].

According to findings, specific families of P450 found in some species of fungi originated by paralogous evolution of member P450s, allowing the organism to adapt to various ecological niches, including the colonization of plant material [28]. Therefore, P450 family growth is feasible due to P450 member duplication in an organism [29,30]. The existence of several families and clans found in the eight chosen *Aspergillus* species shows expansions of the fungal CYPs families. We believe this is a result of the evolution of several fungal properties, including pathogenicity. The results also showed unique CYP families and clans in some chosen *Aspergillus* species. We believe that this has a role in the host specificity of the fungus species to plants or animals. According to Rampersad [31], distinct CYPs found in various fungus species may account for the host specificity of each fungus species to a particular plant or animal.

Our study further reveals that irrespective of the *Aspergillus* spp., the endoplasm reticulum turned out to be the dominant organelle for the localization of their cyp protein genes. Based on how electrons are transferred to their catalytic site from NAD (P) H, cyps have been categorized into four classes, of which fungi and other eukaryotes are most commonly found in class II enzymes, with the endoplasmic reticulum being the dominant subcellular component of their cyps [32]. The cyps protein genes of *A. cabonarius* and *A. terreus* were the most distributed across the subcellular components. The localization of P450s in six cell organelles (cytoplasm, endoplasm reticulum, peroxisome, microsome, cell membrane, and plasma membrane) of all the queried *Aspergillus* spp. indicates the varying significant roles played by these genes in the *Aspergillus* genus. Fungal species as part of organisms possessing the Class II enzymes are involved in highly diverse functions, including biosynthesis of secondary metabolites (mycotoxins), sterols of membranes, lipid metabolism, and detoxification of xenobiotics and phytoalexins; therefore, their cyps may be localized in more than one subcellular component [32,33].

In response to the surrounding external biotic and abiotic factors, *Aspergillus* species, like other fungi, are known to bio-synthesize some bioactive molecular compounds referred to as secondary metabolites (SMs), which are maybe either beneficial or harmful to their hosts and environment depending on the species as part of their life cycle [34]. The specific genes responsible for producing these SMs are most often positioned so closely as a cluster [35,36]. Given the significant role played by CYP450 enzymes in the metabolic activity of life systems, the present study predicted the engagement of cyp protein genes of *Aspergillus* species in the formation of three out of four classes of secondary metabolism-related gene clusters and their hybrids commonly found in fungi as reported by Keller et al. [37]. These biosynthesis gene clusters encode enzymes (polyketide synthases, dimethylallyl tryptophan synthases, non-ribosomal peptide synthetases, and terpene cyclases) characterized by multiple modules and domains, which are vital for the scaffold formation of secondary metabolites [38,39,40,41]. The observed abundance of PKS, NRPS-like, and NRPS in *Aspergillus* species agrees with the findings of Cox, [42], de Vries et al. [3], and Drott et al. [43], who reported that PKS and NRPS are the most dominant secondary metabolism-related gene clusters in fungal species from which secondary metabolites (SMs) are synthesized. Similarly, the role of P450 genes in the biosynthesis of mycotoxins in fungal species has been well documented [32]. Even though these SMs have not been demonstrated to be essential for the growth and development of *Aspergillus* spp. [44], they are significant for the colonization of their environment by serving as growth inhibitors of their competitors and chemical communicating signals [40,45]. Several SMs synthesized by *Aspergillus* species have been identified. For example, lovastatin produced by *A. terreus* is medically useful for regulating cholesterol in humans [35], while many are detrimental, such as aflatoxins complex from *A. niger* and *A. flavus*, gliotoxin by *A. fumigatus*, and Aspyridones by *A. nidulans* [35].

## 5. Conclusions

*Aspergillus* species are among the most commercially significant fungal organisms globally, with use in human health, industry, and agriculture. Understanding the genome-wide annotations of cytochrome P450s in *Aspergillus* species is critical for biosynthetic applications. This study revealed a total of 306 cytochrome P450s found in eight *Aspergillus* species. According to the evolutionary connection, the *Aspergillus* P450s families are divided into 15 clades, with Clade V containing the most significant Cyp families (17.3%). Cyps were divided into 120 families, 64 clans, and their putative functions. Cytochrome P450 families identified as unique to each of the eight *Aspergillus* spp. could be a target to be harnessed either for their management or biosynthesis of important secondary metabolites. P450s were anticipated to be found in 13 different subcellular components; however, the endoplasmic reticulum was the most common site in all eight *Aspergillus* species. The Cyps genes in the *Aspergillus* species were linked to seven secondary metabolism-related gene clusters. The current study’s findings disclosed some pertinent information that may be used for the appropriate management of *Aspergillus* species, which represent a danger to the sustainable production of various vital crops throughout the world. We conclude that elucidating the genome-wide annotations of P450s enzymes in *Aspergillus* species would result in critical prospective biotechnological tools that may be used in industrial, medicinal, and agricultural applications.

## Figures and Tables

**Table 1:** Taxonomic distribution of putative CYPs in eight *Aspergillus* species.

*Aspergillus* Species	Genome Size (Mb)	Number of Predicted Genes	Total Proteins Sequences	Total Cyp Proteins	Protein with Complete Sequences	Family Type	Clan Type	Families with No FCPD Matches
*A. carbonarius* ITEM 5010 v3	36.29	11,624	222	114	35	29	22	1
*A. clavatus* NRRL 1	27.86	9121	145	91	35	28	20	-
*A. flavus* NRRL3357 v1.0	37.75	13,715	231	139	45	36	28	-
*A. fumigatus* A1163	29.21	9916	130	75	32	31	27	2
*A. nidulans* A1146	30.48	10,680	184	103	30	29	20	-
*A. niger* ATCC 13496 v1.0	35.69	12,197	232	133	34	26	19	-
*A. oryzae* RIB40	37.88	12,030	249	140	48	37	22	-
*A. terreus* NIH 2624	29.33	10,406	183	101	47	35	24	-

**Table 2:** Distribution of identified CYP450 families and clans across the eight selected *Aspergillus* species.

			*Aspergillus* spp.								
Clan	CYP Proteins in Clan	Families	*A. Carbonarius*	*A. Clavatus*	*A. Flavus*	*A. Fumigatus*	*A. Nidulans*	*A. Niger*	*A. Oryzae*	*A. Terreus*	Total
CYP51	2	Cyp51	-	2	-	-	-	-	-	-	2
CYP52	15	Cyp52	-	1	-	-	-	1	1	3	6
		Cyp539	-	-	-	-	-	-	-	1	1
		Cyp584	-	-	1	-	1	1	1	-	4
		Cyp655	-	-	-	-	1	-	-	-	1
		Cyp5087	-	1	-	1	-	-	1	-	3
CYP53	4	Cyp53	1	-	-	-	-	-	1	2	4
CYP54	6	Cyp602	-	1	-	-	-	-	-	-	1
		Cyp503	1	1	-	-	1	1	-	-	4
		Cyp5085	-	-	-	1	-	-	-	-	1
CYP55	2	Cyp55	-	-	-	-	-	1	-	1	2
CYP56	3	Cyp56	1	-	-	-	-	-	-	-	1
		Cyp661	-	-	-	1	-	-	-	-	1
		Cyp5099	-	-	-	-	-	-	1	-	1
CYP58	29	Cyp58	1	-	1	-	-	2	3	3	10
		Cyp551	-	1	-	-	-	-	-	-	1
		Cyp680	1	-	-	-	1	1	-	-	3
		Cyp681	-	1	-	-	-	-	-	-	1
		Cyp682	-	-	1	1	2	1	1	1	7
		Cyp5094	-	1	-	-	-	-	1	-	2
		Cyp5095	1	-	-	-	-	-	1	-	2
		Cyp5096	1	-	-	-	-	-	1	-	2
		Cyp5105	-	-	-	-	-	-	1	-	1
CYP59	8	Cyp59	1	-	2	-	-	1	-	-	4
		Cyp586	-	-	-	1	-	-	-	-	1
		Cyp587	-	-	-	-	-	-	-	1	1
		Cyp662	-	-	-	-	1	-	-	1	2
CYP61	6	Cyp61	-	1	2	-	-	-	-	3	6
CYP62	4	Cyp62	1	-	1	-	-	-	-	1	3
		Cyp684	-	-	-	-	1	-	-	-	1
CYP65	18	Cyp60	-	-	1	-	-	-	1	-	2
		Cyp65	4	2	-	-	-	2	2	2	12
		Cyp567	-	1	-	-	1	-	-	-	2
		Cyp5117	-	-	-	-	-	-	2	-	2
CYP68	12	Cyp68	-	1	1	-	-	2	2	1	7
		Cyp595	-	-	-	-	-	-	-	1	1
		Cyp650	-	-	-	-	1	-	-	-	1
		Cyp5061	-	1	1	-	-	-	-	-	2
		Cyp5073	-	-	-	-	1	-	-	-	1
CYP504	5	Cyp504	2	1	-	-	-	-	1	1	5
CYP505	6	Cyp505	-	2	1	-	-	-	-	-	3
		Cyp541	1	-	-	1	1	-	-	-	3
CYP507	1	Cyp535	-	-	1	-	-	-	-	-	1
CYP512	1	Cyp512	-	-	-	-	-	-	-	1	1
CYP526	2	Cyp526	-	1	-	-	-	-	-	1	2
CYP530	17	Cyp530	-	-	-	1	1	-	-	-	2
		Cyp619	1	2	-	-	1	-	-	1	5
		Cyp663	1	-	-	-	-	-	-	-	1
		Cyp665	-	-	-	-	-	-	-	1	1
		Cyp5068	-	1	-	1	-	-	-	1	3
		Cyp5069	-	-	1	-	-	-	-	-	1
		Cyp5093	-	1	1	-	-	-	-	1	3
		Cyp5098	-	-	-	-	-	-	1	-	1
CYP531	23	Cyp531	-	2	-	1	1	-	2	2	8
		Cyp532	-	-	1	-	-	-	-	-	1
		Cyp631	-	-	-	-	-	-	1	-	1
		Cyp674	-	-	-	-	1	-	-	-	1
		Cyp675	-	-	-	-	1	-	-	-	1
		Cyp5077	-	-	-	-	1	-	-	1	2
		Cyp5078	-	-	-	-	-	-	-	1	1
		Cyp5080	2	-	1	1	1	1	1	-	7
		Cyp5104	1	-	-	-	-	-	-	-	1
CYP533	9	Cyp64	-	-	-	-	-	-	1	-	1
		Cyp620	-	1	2	-	1	2	1	1	8
CYP537	6	Cyp537	2	-	1	-	-	-	-	1	4
		Cyp577	-	1	-	-	-	-	-	1	2
CYP540	2	Cyp540	-	-	-	-	-	1	1	-	2
CYP547	11	Cyp547	-	-	-	1	-	-	-	-	1
		Cyp617	-	2	1	2	-	2	-	2	9
		Cyp5070	-	-	1	-	-	-	-	-	1
CYP548	12	Cyp548	1	2	2	1	-	1	2	1	10
		Cyp5114	-	-	1	-	-	-	1	-	2
CYP550	6	Cyp660	-	1	2	1	-	-	2	-	6
CYP566	1	Cyp566	-	-	-	1	-	-	-	-	1
CYP572	5	Cyp573	1	-	1	1	-	1	-	1	5
CYP574	16	Cyp628	-	-	1	-	-	-	1	-	2
		Cyp669	1	-	-	-	-	1	-	-	2
		Cyp670	1	-	-	-	-	1	-	-	2
		Cyp671	1	-	1	-	1	2	1	-	6
		Cyp5076	-	-	1	1	-	1	-	1	4
CYP576	2	Cyp576	1	-	-	-	-	-	-	1	2
CYP578	8	Cyp578	-	-	2	1	-	2	1	2	8
CYP589	5	Cyp5075	1	-	2	-	-	-	2	-	5
CYP590	1	Cyp590	1	-	-	-	-	-	-	-	1
CYP603	1	Cyp603	-	1	-	-	-	-	-	-	1
CYP613	6	Cyp613	-	-	1	-	-	-	2	1	4
		Cyp685	-	-	-	-	-	-	-	2	2
CYP630	4	Cyp630	-	-	-	1	1	1	1	-	4
CYP645	1	Cyp645	-	-	-	1	-	-	-	-	1
CYP646	3	Cyp646	-	-	-	-	1	2	-	-	3
CYP648	1	Cyp648	-	-	-	-	1	-	-	-	1
CYP653	4	Cyp653	1	-	1	1	-	-	-	-	3
		Cyp654	-	-	-	-	1	-	-	-	1
CYP659	2	Cyp5090	-	-	-	1	-	-	-	-	1
		Cyp5111	-	-	1	-	-	-	-	-	1
CYP664	1	Cyp664	-	-	-	-	1	-	-	-	1
CYP666	3	Cyp666	-	-	-	-	-	1	1	1	3
CYP672	1	Cyp672	-	-	-	1	-	-	-	-	1
CYP673	1	Cyp673	-	-	-	-	-	-	1	-	1
CYP677	1	Cyp5064	-	-	1	-	-	-	-	-	1
CYP678	1	Cyp678	-	-	-	-	1	-	-	-	1
CYP683	1	Cyp683	-	-	-	-	1	-	-	-	1
CYP687	1	Cyp687	-	-	-	-	1	-	-	-	1
CYP698	1	Cyp698	-	-	-	-	-	-	1	-	1
CYP5042	4	Cyp5042	-	-	3	-	-	-	1	-	4
CYP5052	1	Cyp5052	-	-	-	1	-	-	-	-	1
CYP5063	1	Cyp5063	-	-	-	1	-	-	-	-	1
CYP5081	1	Cyp5081	-	-	-	1	-	-	-	-	1
CYP5083	1	Cyp5083	1	-	-	-	-	-	-	-	1
CYP5084	1	Cyp5084	-	-	-	1	-	-	-	-	1
CYP5089	2	Cyp5089	-	1	-	-	-	1	-	-	2
CYP5101	1	Cyp5101	-	-	1	-	-	-	-	-	1
CYP5071	3	Cyp5106	1	-	1	-	-	-	1	-	3
CYP5110	1	Cyp5110	-	1	-	-	-	-	-	-	1
CYP5116	1	Cyp5116	-	-	-	-	-	1	-	-	1
Absent		Cyp468	-	-	-	1	-	-	-	-	1
Absent		Cyp5051	-	-	-	1	-	-	-	-	1
Absent		Cyp5125	1	-	-	-	-	-	-	-	1
Total CYP families in each *Aspergillus* sp.			35	35	45	32	30	34	48	47	**306**

**Table 3:** Phylogenetic clustering of Cytochrome P450 families and clans amongst eight *Aspergillus* spp.

Phylogenetic Clade	Sequence Entry	CYP Families	CYP Clans	Putative Functions
I	46	Cyp65, Cyp60, Cyp5117, Cyp567, Cyp672, Cyp5083, Cyp566, Cyp548, Cyp5114, Cyp671, Cyp628, Cyp670, Cyp669, Cyp5076, Cyp578	CYP65, CYP672, CYP5083, CYP566, CYP548, CYP574, CYP578	Primary metabolism, Secondary metabolism
II	5	Cyp5084, Cyp680, Cyp673, Cyp535, Cyp567	CYP5084, CYP673, CYP58, CYP65, CYP507	Secondary/xenobiotic metabolism
III	7	Cyp630, Cyp53	CYP630, CYP53	Xenobiotic/primary metabolism
IV	27	Cyp573, Cyp5080, Cyp5104, Cyp675, Cyp674, Cyp531, Cyp5077, Cyp631, Cyp5078, Cyp532	CYP572, CYP531	Xenobiotic metabolism
V	53	Cyp578, Cyp577, Cyp537, Cyp683, Cyp5089, Cyp53, Cyp62, Cyp684, Cyp678, Cyp5064, Cyp680, Cyp5095, Cyp5096, Cyp682, Cyp551, Cyp5094, Cyp58, Cyp681, Cyp5105, Cyp5097	CYP578, CYP58, CYP537, CYP683, CYP53, CYP62, CYP678, CYP677, CYP5097, CYP5089	Secondary/xenobiotic Metabolism
VI	3	Cyp5081, Cyp576	CYP5081, CYP576,	
VII	8	Cyp59, Cyp587, Cyp586, Cyp662	CYP59	Xenobiotic metabolism
VIII	17	Cyp617, Cyp547, Cyp5070, Cyp526, Cyp540, Cyp468	CYP547, CYP540, CYP526,	Primary/xenobiotic metabolism
IX	30	Cyp5075, Cyp5106, Cyp590, Cyp666, Cyp56, Cyp5099, Cyp661, Cyp655, Cyp5087, Cyp52, Cyp5051, Cyp539, Cyp548, Cyp584	CYP5075, CYP56, CYP5071, CYP666, CYP52, CYP590, Cyp548	Secondary metabolism
X	7	Cyp613, Cyp686, Cyp685	CYP613	Xenobiotic metabolism
XI	38	Cyp5098, Cyp5052, Cyp5068, Cyp663, Cyp5063, Cyp5097, Cyp5069, Cyp5093, Cyp665, Cyp530, Cyp664, Cyp619, Cyp5042, Cyp620, Cyp64, Cyp504	CYP530, CYP533, CYP504, CYP5052, CYP5097, CYP5063, CYP5042, CYP664	Xenobiotic metabolism
XII	10	Cyp61, Cyp5116, Cyp55, Cyp51	CYP61, CYP55, CYP51, CYP5116	Primary metabolism
XIII	45	Cyp660, Cyp5090, Cyp5101, Cyp5111, Cyp5100, Cyp5106, Cyp646, Cyp5110, Cyp653, Cyp503, Cyp698, Cyp602, Cyp5093, Cyp654, Cyp5125, Cyp503, Cyp512, Cyp5085, Cyp648, Cyp603, Cyp595, Cyp68, Cyp5061, Cyp5074, Cyp5073, Cyp650	CYP550, CYP659, CYP5101, CYP5070, CYP54, CYP639, CYP648, CYP646, CYP68, CYP5110, CYP603, CYP512, CYP530, CYP653, CYP698, CYP608,	Secondary/xenobiotic Metabolism
XIV	7	Cyp505, Cyp541	CYP505, CYP5125	Primary metabolism
XV	3	Cyp682, Cyp645, Cyp687	CYP645, CYP687, CYP58	Secondary/xenobiotic metabolism

## Data Availability

Data used for all analyses supporting reported results, and their links have been included in the manuscript.

## Supplementary Materials

The following are available online at https://www.mdpi.com/article/10.3390/life12030451/s1, supplementary data file S1: Total cytochromeP450 gene IDs of 8 Aspergillus species downloaded from JGI, supplementary data file S2: Validated CytochromeP450 gene IDs of 8 *Aspergillus* species used for the analysis, supplementary data file S3: Validated cytochrome P450 protein sequences (306) from 8 *Aspergillus* species used.
